# Draft Genome Sequence of the First South Korean Clinical Isolate of Burkholderia pseudomallei, H0901

**DOI:** 10.1128/genomeA.00336-18

**Published:** 2018-04-26

**Authors:** Yong-Woo Shin, Myung-Min Choi, Jeong-Hoon Chun, Jae-Yon Yu, Dae-Won Kim, Gi-eun Rhie

**Affiliations:** a Division of High-Risk Pathogens, Center for Laboratory Control of Infectious Diseases, Korea Centers for Disease Control and Prevention, Cheongju, South Korea

## Abstract

We report here the draft genome sequence of Burkholderia pseudomallei H0901. This strain was isolated in 2003 from the first melioidosis patient in South Korea.

## GENOME ANNOUNCEMENT

Burkholderia pseudomallei is a Gram-negative bacterium that can cause melioidosis, a severe infectious disease (1). The pathogen is found mainly in southeastern Asia, northern Australia, and on various Pacific and Indian Ocean islands, where melioidosis is endemic (2, 3). Diagnosis of melioidosis is confirmed through isolation of the bacterium from a sample taken from the blood, sputum, skin lesions, abscesses, or urine of patients (4). The melioidosis patient may display various conditions, ranging from subclinical to overwhelming protean manifestations resembling chronic and acute bacterial infections (5). As treatment of B. pseudomallei infections can be lengthy, accurate and rapid diagnosis of melioidosis is necessary for efficacious antimicrobial therapy (4, 6).

In 2003, we encountered the first case of melioidosis in South Korea. The patient was a 47-year-old male with septicemia and septic pneumonia who had a history of traveling to Malaysia and suffered from diabetes mellitus (5). The bacterial isolate from the blood of the patient was identified as B. pseudomallei based on DNA sequencing of the 16S rRNA gene and on biochemical tests (7).

For the draft genome sequence of B. pseudomallei H0901, genomic DNA was isolated from B. pseudomallei H0901 grown in Ashdown’s medium with gentamicin for 24 h at 37°C (8). The paired-end sequencing library was prepared using a genomic DNA prep kit (Qiagen, USA). A MiSeq instrument (Illumina, San Diego, CA) was used as the sequencing platform. A total of 7,956,141 reads were obtained by whole-genome sequencing, and fold coverage was 235. Finally, 142 contigs were assembled from all reads in CLC Genomic Workbench (CLCbio, version 10.0) using thresholds with a minimum of 500 bp (9). The number of *N*_50_ contigs was 88,367. The draft genome of the H0901 strain consisted of 7,001,996 bp of nucleotides (G+C content, 68.2%). The NCBI Prokaryotic Genome Annotation Pipeline (PGAP) predicted 6,836 protein-coding genes, 2 rRNAs, 54 tRNAs, and 407 pseudogenes.

From 2003 to 2014, 11 cases of melioidosis were reported in South Korea (10). Each patient had a history of traveling to southeastern Asian countries, and melioidosis is still considered an imported infectious disease in South Korea. However, due to the increase of traffic to countries where melioidosis is endemic and the increase of environmental temperature by global warming, it is possible that the occurrence of melioidosis is increasing in South Korea. In this study, we report the draft genome sequence of B. pseudomallei H0901, isolated from the first melioidosis patient in South Korea. B. pseudomallei H0901 will be used as a reference strain for the confirmatory identification of B. pseudomallei in the Korea Centers for Disease Control and Prevention.

### Accession number(s).

This whole-genome shotgun project was deposited in DDBJ/EMBL/GenBank under the accession number NEGN00000000. The version described in this paper is the second version, NEGN02000000.
